# Advances in Understanding the Phenomena and Processing in Audiovisual Speech Perception

**DOI:** 10.3390/brainsci13091345

**Published:** 2023-09-20

**Authors:** Kaisa Tiippana

**Affiliations:** Department of Psychology and Logopedics, University of Helsinki, 00014 Helsinki, Finland; kaisa.tiippana@helsinki.fi

The Special Issue entitled “Advances in Understanding the Phenomena and Processing in Audiovisual Speech Perception” attracted a variety of articles written by prominent authors in the field. The articles include research reports, reviews and opinions addressing audiovisual, auditory, visual and cross-modal speech perception.

With regard to how multisensory perception changes with age, Pepper and Nuttall [1] present a narrative review of findings from various experimental paradigms. They propose that changes that occur with aging in multisensory processing (including audiovisual speech) may be linked with balance control via similar mechanisms. Those are suggested to be related to declining selective attention with alpha-band oscillations as a neural correlate.

The early development of infant language learning in the context of multisensory processing is addressed in the review by Birulés, Goupil, Josse and Fort [2]. They first acknowledge that laboratory studies have provided a wealth of findings contributing to the knowledge of AV speech perception and processing in infants. They then point out the need to link the contributions of laboratory studies with research on everyday natural interactions. This will be a crucial future step, not only in infant studies, but also in general AV speech perception research.

Learning is an important topic in speech research. It is still not well understood how A, V and AV speech are learned. Studies on the effects of different types of training and experience provide much-needed contributions to this knowledge. As far as long-term (lifetime) experience is concerned, Kawase, Davis and Kim [3] found that a familiar speech rhythm (when a speaker talks in their mother tongue) benefits AV speech perception in noise; meanwhile, an unfamiliar speech rhythm (when a speaker has a strong foreign accent) does not.

Bernstein, Auer and Eberhardt [4] show that the learning of novel spoken words is based on different cues in audition and vision, both within each modality and cross-modally. This suggests that a single optimal training paradigm does not exist. Instead, learning depends on the sensory modality, as well as the training task. This study sheds light on why A/V/AV speech training has often met with limited success. Furthermore, the authors offer a theoretical account of perceptual learning of speech (see also [5]).

In their opinion paper, Moradi and Rönnberg [6] present a hypothesis according to which brief exposures to audiovisual speech, gradually increasing in duration, facilitate the subsequent auditory processing of speech, while auditory exposures do not. They review findings supporting this. They also present a theoretical framework for the underlying mechanism and suggest that presenting AV speech segments before A speech recognition boosts performance by tuning the phonological representations in long-term memory. In line with this view, though in a different context, Zadoorian and Rosenblum [7] found in their experimental study that AV training with talking and static faces improved voice recognition compared to auditory training.

A different way of boosting speech processing is cued speech (CS), where manual gestures are added to AV speech to provide additional visual phonetic cues. Caron et al. [8] studied whether experience in using CS influences the neural processing of speech. In an EEG study, they first replicated the known N1 and P2 attenuation of auditory evoked potentials by visual speech. Their novel finding is that CS had different effects on auditory potentials in naïve young adults and experienced CS users, showing that learning novel visual cues can modulate auditory speech processing.

Audiovisual speech perception has also been studied in various special populations. The current issue contains two articles related to autism spectrum disorder (ASD). In the first EEG study, adults with a range of autism-like traits were studied, and higher levels of traits were found to be associated with attenuated P3 responses to AV speech (Harwood et al. [9]). In the other study, groups of ASD and typically developing school-aged children were investigated, and individual differences in P2 amplitude attenuation were found to be positively associated with expressive vocabulary through receptive vocabulary (Dunham-Carr et al. [10]). These findings add to the evidence of the atypical processing of AV speech in individuals with traits associated with ASD. A similar conclusion was made with respect to schizophrenia, based on findings that the recognition of AV speech in noise was poorer in participants with schizophrenia compared to those without schizophrenia and that N1 amplitude attenuation correlated with increasing schizophrenia symptoms (Ghaneirad et al. [11]).

The abovementioned articles deal with natural speech in the sense that AV signals are congruent syllables or words. Instead, in the well-known AV speech illusion—the McGurk effect—an incongruent visual consonant alters the speech percept [12]. For example, in the most classical McGurk stimulus, the A consonant is /b/, the V consonant is /g/ and the most common illusory percept is often /d/. This illusion has been used as a tool to study the integration of audiovisual speech in hundreds of studies since its discovery by McGurk and MacDonald in 1976 [12]. However, only two studies in this issue deal with the McGurk effect. This is not surprising considering recent criticism claiming that the McGurk effect cannot reflect speech perception in real life since it is an artificial stimulus and an unstable illusion [13,14,15].

Iqbal et al. [16] contribute to the topical discussion on the nature of the McGurk effect by proposing that it arises when both A and V speech are ambiguous and integration does not occur, resulting in a default A response. They show that when the A consonant is removed from the stimulus, participants still often respond /d/, suggesting that when the auditory signal is ambiguous, the default A percept is /d/. In addition, V/g/ is known to be confusable with /d/ (e.g., [17]). Consequently, they argue that the McGurk effect occurs when integration fails and perception defaults to the /d/ phoneme.

Since the findings regarding cross-language effects on the McGurk effect have been mixed, Tiippana et al. [18] wanted to compare Finnish and Japanese speakers and listeners. The results showed that language had little effect. Instead, there were large differences between individual speakers in the McGurk effect, as well as in the perception of unisensory stimulus components. They concluded that A and V stimulus features should be characterized to clarify how they contribute to the illusion.

This Special Issue gives a view into current audiovisual speech perception research. The field is active in both established research lines and new directions. It is amazing that some issues remain an enigma despite decades of research (e.g., how the McGurk effect arises). Fortunately, further evidence keeps accumulating, and theories and models are being developed to account for the processing mechanisms underlying AV speech perception.

Multisensory learning is a theme that is being studied from many angles from short-term training to lifetime development. Speech perception is an active process molded by experiences. A notable contribution of the current issue is the experimental finding revealing which different cues are used in learning A and V speech, together with a theory providing an explanation for this finding [4]. Importantly, the theory makes it possible to make testable predictions for future studies.

Future directions arising from the Special Issue include the development of theories and models, since they are crucial for understanding the perception and processing of audiovisual speech. Combining knowledge of unisensory and audiovisual speech processing is needed for a complete picture. Regarding speech in general, the bulk of research thus far has concentrated on phoneme perception. Lately, an increasing number of studies have started to use words and sentences as stimuli. The trend is towards more naturalistic speech situations. Future research should aim to bridge the gap between real-life situations, which typically involve interaction (continuous speech or conversation) in a multisensory environment, and the existing large knowledge base of speech segment perception.
